# Cutaneous hypersensitivity reactions to freshwater cyanobacteria – human volunteer studies

**DOI:** 10.1186/1471-5945-6-6

**Published:** 2006-04-04

**Authors:** Ian Stewart, Ivan M Robertson, Penelope M Webb, Philip J Schluter, Glen R Shaw

**Affiliations:** 1 National Research Centre for Environmental Toxicology, University of Queensland, 39 Kessels Road, Coopers Plains, QLD 4108, Australia; 2 School of Population Health, University of Queensland, Herston Road, Herston, QLD 4006, Australia; 3 Cooperative Research Centre for Water Quality and Treatment, PMB 3, Salisbury, SA 5108, Australia; 4 Department of Dermatology, Royal Brisbane and Women's Hospital, Butterfield Street, Herston, QLD 4029, Australia; 5 Queensland Institute of Medical Research, 300 Herston Road, Herston, QLD 4006, Australia; 6 Faculty of Health and Environmental Sciences, Auckland University of Technology, Private Bag 92006, Auckland 1020, New Zealand; 7 School of Public Health, Griffith University, University Drive, Meadowbrook, QLD 4131, Australia

## Abstract

**Background:**

Pruritic skin rashes associated with exposure to freshwater cyanobacteria are infrequently reported in the medical and scientific literature, mostly as anecdotal and case reports. Diagnostic dermatological investigations in humans are also infrequently described. We sought to conduct a pilot volunteer study to explore the potential for cyanobacteria to elicit hypersensitivity reactions.

**Methods:**

A consecutive series of adult patients presenting for diagnostic skin patch testing at a hospital outpatient clinic were invited to participate. A convenience sample of volunteers matched for age and sex was also enrolled. Patches containing aqueous suspensions of various cyanobacteria at three concentrations were applied for 48 hours; dermatological assessment was made 48 hours and 96 hours after application.

**Results:**

20 outpatients and 19 reference subjects were recruited into the study. A single outpatient produced unequivocal reactions to several cyanobacteria suspensions; this subject was also the only one of the outpatient group with a diagnosis of atopic dermatitis. No subjects in the reference group developed clinically detectable skin reactions to cyanobacteria.

**Conclusion:**

This preliminary clinical study demonstrates that hypersensitivity reactions to cyanobacteria appear to be infrequent in both the general and dermatological outpatient populations. As cyanobacteria are widely distributed in aquatic environments, a better appreciation of risk factors, particularly with respect to allergic predisposition, may help to refine health advice given to people engaging in recreational activities where nuisance cyanobacteria are a problem.

## Background

Cyanobacteria, commonly but erroneously known as blue-green algae, are common inhabitants of freshwater lakes and reservoirs throughout the world. Under favourable conditions certain cyanobacteria can dominate the phytoplankton within a waterbody and undergo mass developments, known as blooms. Public health concerns arise because many nuisance cyanobacteria can produce potent toxins. Anecdotal and case reports have documented skin rashes, often described as intensely pruritic, associated with contact exposure to cyanobacteria. While there are relatively few references in the scientific and medical literature since these reports began in 1949, under-diagnosis of cyanobacteria-associated illness was suggested by Schwimmer & Schwimmer [1] in 1968, a suspicion that probably holds today. Most reports of cyanobacteria-associated skin eruptions describe recreational or occupational exposure [2], however there are anecdotal reports of skin rashes related to water treatment failures and subsequent presence of cyanobacterial products in reticulated supplies. In these instances, skin rashes were reported after showering or bathing [3,4]. "Several" people experienced acute dermatitis, as well as gastrointestinal symptoms, after drinking water from a riverine source affected by a cyanobacteria bloom in Portugal [5].

Skin patch testing is a routine diagnostic procedure in dermatology clinics worldwide, and testing with cyanobacterial preparations was first reported in the USA in 1953 to investigate a water contact-related seasonal dermatitis in a girl aged six years. Strong positive reactions to various extracts of an *Anabaena *sp. dominant bloom sample were observed on the child but none of 25 healthy control subjects [6].

In a study of volunteers to investigate irritant reactions, Pilotto *et al *[7] reported that 20–24% of subjects reacted to cyanobacterial test patches, and 23% of subjects responded to negative control patches. After excluding subjects who responded to the negative controls, 11–15% of subjects responded to cyanobacteria. No dose-response relationships were reported.

Anecdotal and case reports in the medical and scientific literature do not provide convincing descriptions of mass outbreaks of cutaneous symptoms associated with recreational or occupational exposure to planktonic cyanobacteria. Rather, the picture is of isolated events affecting individuals or small numbers of people [2]. An epidemiological study to investigate the occurrence of acute symptoms did not find a statistically significant difference in the reporting of cutaneous symptoms across groups exposed to different levels of planktonic cyanobacteria in recreational waters. The small number of subjects that reported skin ailments after bathing in cyanobacteria-affected waters mostly rated the severity of symptoms as mild [8]. Taken together, these findings suggest that nuisance planktonic cyanobacteria are not commonly present at irritant concentrations in inland recreational waters, unlike the marine filamentous cyanobacterium *Lyngbya majuscula*, which is known to produce dermally-active toxins and has been linked to mass outbreaks of acute dermatitis involving hundreds of individuals, with high proportions of exposed individuals being affected [9].

The purpose of this study was to assess the propensity for a range of cyanobacterial suspensions to induce cutaneous irritant and hypersensitivity reactions in dermatology outpatients and a reference group of volunteers matched for age and sex. We wished to determine whether threshold doses that induce reactions in the reference group, if indeed such reactions occur in this group, are lower in individuals with an active history of cutaneous symptoms. Irritant and hypersensitivity reactions would be determined both qualitatively and quantitatively, and the cyanobacteria would be characterised in terms of species (or genera if speciation were not possible), doses to be applied to the skin, and the presence or absence of known toxins.

## Methods

### Study participants, patch application and reading

A consecutive series of adults aged 18 to 65 years presenting for diagnostic skin patch testing at the Royal Brisbane and Women's Hospital dermatology outpatient clinic between March 2002 and November 2003 was invited to participate in the study – provided they met study inclusion criteria – until 20 were recruited. A convenience sample of volunteers was recruited via notices posted at three university sites and by word of mouth for the reference group. Patients and reference subjects were matched by sex and, where possible, by age using 5 year age bands. Routine exclusion criteria for elective patch testing applied to this study: persons with infectious dermatoses, widespread acne, traumatic lesion or excess hair on their back. Pregnant women were also excluded.

Study subjects were asked to complete a simple questionnaire that requested basic demographic details (age, sex), history of allergic illness (asthma, hay fever, eczema, urticaria), relevant medications and a description of any freshwater-related dermatoses [[10] (Appendix 4)].

The skin patch testing procedure uses a series of shallow aluminium chambers (Finn chambers), 8 mm internal diameter, 0.5 mm depth, into which test materials are placed, either impregnated onto discs of filter paper or mixed in petrolatum [11]. Test material is placed in each chamber, and the Finn chamber strips are fixed on the skin with non occlusive, non-allergenic and non-irritant adhesive tape. For this study a clinic nurse prepared the skin of each subject's back with acetone, and patches were applied to the skin. Study subjects were instructed to keep their back dry, i.e. bathe but not shower, and to refrain from sport or vigorous activity that might lead to frank perspiration, with resultant separation of Finn chamber strips from the skin. Patches were then removed after 48 hours. The clinic nurse marked the position of each Finn chamber with a permanent marker pen; after allowing adhesive tape-related erythema to subside, patch test sites were read by a dermatologist after 48 and approximately 96 hours. Patch sites were scored according to the key in Table 1.

Dermatology clinic workers were blinded to the identity of test materials (patch series columns) but not to test concentrations (patch series rows) because we thought that identification of concentration-dependent (i.e. dose-response) reactions to any particular test suspension series would assist in the differentiation of irritant and hypersensitivity responses. Clinic workers were not blinded to the status of study subjects as either patients or non-patients.

Ethical approvals for this study and amendments were granted by the Royal Brisbane Hospital Health Service District's Human Research Ethics Committee, protocol number 2001/151, and the University of Queensland's Medical Research Ethics Committee, clearance number 2002000099.

### Patch test materials

Six cyanobacterial suspensions, two cyanobacterial lipopolysaccharide extracts and one eukaryotic algal suspension were tested, each at three concentrations. Sodium dodecyl sulfate was used as a positive irritant control. The test materials and measured cyanotoxin concentrations are listed in Table 2.

### Culturing of cyanobacterial isolates; preparation of stock suspensions

Cyanobacteria isolates were non-axenic laboratory cultures grown in sterile inorganic media in an illuminated growth chamber at 28°C with a 14:10 light/dark cycle. Culture vessels were aerated with aquarium pumps and air-stones connected by PVC tubing; air was delivered through 0.45 μm Millipore^® ^filters, and all culture vessels and air delivery components distal to the filter (tubing, weights and air-stones) were sterilised prior to use by steam autoclaving or Sterrad^® ^hydrogen peroxide plasma sterilisation (the latter for heat labile plastics).

*M. aeruginosa *and *Planktothrix *sp. cultures were grown in 20L batch cultures; *M. aeruginosa *cells were harvested by placing the culture vessel in a darkened cupboard overnight. This caused cells to rise to the surface of the vessel where they were aspirated with a syringe and PVC tubing. *Planktothrix *sp. is a filamentous cyanobacterium, so was easily harvested by plucking it in several continuous sheets from the vessel walls and aeration tubing. *C. raciborskii *was produced by continuous culture adapted from the method of Court *et al *[12] and cells were concentrated by centrifugation in 750 mL centrifuge bottles, then decanting and discarding media. Cells were double-washed by repeat suspension in de-ionised water followed by centrifugation. 250 mL of *C. vulgaris *culture was purchased from CSIRO Hobart, which after double washing yielded sufficient cellular material for this work. Harvested cells were lyophilised, powdered with a domestic coffee grinder and stored at room temperature in air-tight containers.

Stock preparations were made by suspending 25 mg lyophilised cells in 10 mL Milli-Q^® ^filtered water to produce 0.25% w/v suspensions. These were steeped overnight at 4°C. Cell integrity was disrupted by subjecting each suspension to ultrasonic pulsing for 30 seconds, using a Branson Ultrasonics Sonifier 450 instrument. From each 0.25% preparation 1 mL was added to 4 mL Milli-Q^® ^water to produce the 0.05% suspension, and 0.5 mL of that preparation was added to 4.5 mL water for the 0.005% suspension. All suspensions were stored at -20°C.

Lipopolysaccharide (LPS) solutions were prepared from LPS isolated and purified with a hot phenol method and ultracentrifugation, per procedures No. 4: Bacterial lipopolysaccharides – Gram-negative (modified Westphal) and No. 27: Purification of lipopolysaccharide (modified Westphal) ([13], (pp.3-4, 31-2)), from the process described by Westphal & Jann [14]. LPS concentrations were based on the percentage yield from cyanobacterial whole cells they were extracted from:

• *M. aeruginosa *LPS was 0.51% of dry cell weight, so the maximum concentration of LPS for skin patch testing was (5.1 × 10^-3^) × 0.25% w/v, i.e. 13 ppm. Intermediate and low concentrations were prepared by diluting the 13 ppm concentration as described above to give 3 ppm and 300 ppb concentrations.

• *C. raciborskii *LPS was 1.25% of dry cell weight, so the three concentrations of this LPS were 30 ppm, 6 ppm and 600 ppb.

Sodium dodecyl sulfate was prepared at concentrations of 2.0%, 0.4% and 0.04% (w/v in Milli-Q^® ^water) and stored at -20°C.

Microcystins, saxitoxins and cylindrospermopsin were quantified at Queensland Health Scientific Services, Brisbane. These data are included in Table 2; methodology and instrumentation were as outlined in the accompanying paper by Stewart *et al *[15].

### Calculation of cyanotoxin doses applied to skin

Cyanobacterial cell suspensions were applied to filter paper discs that fit into each Finn chamber. A plastic transfer pipette was used to saturate each disc; one or two drops – mostly one drop – are sufficient to saturate the disc. The volume of two transfer pipette drops was measured with an air displacement pipette and found to be 65 μL. Doses were calculated from the maximum concentration (0.25% w/v), then one fifth and one fiftieth of the maximum dose, representing the 0.05% w/v and 0.005% w/v concentrations, were added to estimate the total cutaneous dose for an average 65 kg subject.

### Statistical analysis

Comparisons of categorical variables were undertaken using Fisher's exact test. A p-value < 0.05 was used to define statistical significance and all calculations were conducted using SPSS v13.0. Investigation into the incidence of reactions and threshold concentrations of cyanobacteria, adjusted for covariates including reported history of asthma, urticaria or hay fever was planned but not done because only a single subject developed unequivocal reactions to patches containing cyanobacteria.

## Results

From the consecutive series of outpatients approached, two declined to participate (one of each sex) and one female who agreed to participate was not included due to an administrative oversight. All outpatients were matched to reference subjects by sex (females: *n *= 12; males: *n *= 8). Matching was also done by age (± 5 years), except for three older outpatient subjects (aged 54, 56 and 62 years).

Responses to the questionnaire enquiry regarding a previous history of allergic illness and acute or chronic skin reactions are summarised in Table 3. Outpatients reported significantly more life-time and recent eczema or dermatitis diagnoses (p = 0.04 and p = 0.01 respectively), and rash of unknown cause within the last two years (p = 0.003) than their reference counterparts.

### Skin patch testing – cyanobacterial and algal suspensions

Subjects CO10 and PT05 were removed from consideration of summary statistics given in Table 2. Subject CO10 developed a localised folliculitis over four test series sites – one being the SDS series – so 96-hour readings were uninterpretable. The dermatologist noted a general irritant reaction over the patch area. We were unable to recruit another volunteer in her place, thus the study included 19 reference subjects. Subject PT05 developed "angry back", which is a state of skin hyper-reactivity caused by a strong reaction to one or more patch-test allergens, and is associated with false-positive reactions to other test materials [[16] (pp. 16–17)]; another outpatient subject was recruited to replace this subject in the study.

Table 4 shows results of patch test inspections of the cyanobacterial and algal series. Only one of the outpatient group and none of the reference group showed an unequivocal reaction to cyanobacterial preparations. A weak irritant response to an *A. circinalis *patch was seen in another dermatology outpatient subject, and equivocal responses to various patch materials were seen in four patients and four reference subjects.

Estimated cyanotoxin doses applied to each subject are presented in Table 5. Assuming that two drops of cell suspension were required to saturate each Finn chamber filter disc, and also assuming that the entire volume applied to the discs was in contact with subjects' skin, all doses were well below the mammalian i.p. toxic dose.

## Discussion

### Patch-testing of cyanobacteria and *C. vulgaris*

Only one clear response to this skin-patch testing study was seen, from PT19, a male outpatient subject aged 35 years. Interestingly, this subject was also the only one of 20 outpatients with a diagnosis of atopic dermatitis. We did not conduct any statistical analysis of these results, as it is not appropriate to make such comparisons on the basis of a single subject response. This subject developed unequivocal responses to two cyanobacterial isolates, two bloom samples, and probably to *C. vulgaris *as well. There was no evidence of any dose-response effect in the reactions on this subject's skin. Another point of interest in this subject's patch-test results is that reactions developed to the non-toxic Lake Coolmunda *M. aeruginosa *bloom sample, but no reaction was produced by the toxin-producing *M. aeruginosa *isolate. While the Coolmunda bloom sample was largely a monoculture of *M. aeruginosa*, as with many cyanobacteria blooms there were other cyanobacterial species and genera present in smaller amounts. This leaves open the possibility that this subject has demonstrated hypersensitivity reactions to components other than *M. aeruginosa *in the two bloom samples. Subject PT19 also registered positive responses to both patch series containing *C. raciborskii *and cylindrospermopsin (*C. raciborskii *AWT 205 isolate and North Pine Dam bloom sample). This is interesting in light of the findings by Stewart *et al *[15], which demonstrate that *C. raciborskii *and purified cylindrospermopsin are capable of producing irritant reactions and delayed-contact hypersensitivity in mice.

The principal conclusions from this study are that cutaneous responses to cyanobacteria are uncommon, with only one of 39 subjects demonstrating significant cutaneous responses to cyanobacterial suspensions. Given this patient's diagnosis of atopic dermatitis, and reports in the literature which are suggestive of other features of atopy [2], further research into this matter may benefit from more specific entry criteria to allow investigation of atopic individuals. This sole diagnosis of atopy must be interpreted cautiously, however, in that diagnoses were only available for the twenty outpatients. As the reference group did not have a comprehensive medical history taken, we cannot infer presence or absence of atopic subjects within the reference group.

Weak reactions to *C. vulgaris *were seen on the skin of subject PT19, and possibly one other subject (PT04). *C. vulgaris*, a common and widespread eukaryotic alga, was chosen as a reference material; *Chlorella *spp. are reportedly allergenic [17,18], although *C. vulgaris *has been promoted as an allergy preventative and has some anti-inflammatory properties [19]. Acute skin symptoms have been reported from exposure to other freshwater and marine eukaryotic microalgae [20,21].

Considering the single subject response to cyanobacterial patch testing, these data were used to determine sample size estimates that would produce with high probability a statistically significant result. Using nQuery Advisor^® ^4.0 [22], a Fisher's exact test with α = 0.05 two-sided significance level will have 80% power to detect the difference between a Group 1 proportion of 0.050 and a Group 2 proportion of 0.001 when the sample size in each group is 167. A study involving over 300 volunteers would be prohibitively large and expensive; a more targeted approach in future to recruit subjects from more at-risk populations awaits further knowledge of the mechanisms of cyanobacterial toxicity by the cutaneous route.

### History of skin disease, allergy

As anticipated, the outpatient group contained a higher proportion of subjects with cutaneous disease than the reference group (see Table 3). However, the percentage of subjects reporting hay-fever, asthma and urticarial diagnoses was higher in the reference group, although these differences were not statistically significant. To the extent that future research efforts in this field may need to concentrate on those individuals with atopic illness, recruitment from a dermatology outpatient population may not confer any particular advantage over recruitment from the general population.

### Reactions to sodium dodecyl sulfate

Overall, 44% of subjects (n = 17) did not respond to SDS. Some workers have added SDS to their standard allergy patch test series in order to help differentiate between irritant and allergic reactions [23,24]. However, these workers did not appear to have blinded themselves to the location of SDS patches; they were apparently using reactions to SDS as reference irritant responses from which to compare reactions to allergen patches. We suspect that the inclusion of SDS as a positive irritant control may not have been the most appropriate procedure in this diagnostic patch testing study; this matter is discussed further by Stewart [[10] (Chapter 4)].

### Rationale for determining cyanobacteria concentrations in patch test wells

Our initial challenge was to determine appropriate doses of cyanobacteria to apply to human skin. Prior to commencing this human volunteer study, preliminary irritant mouse ear swelling work had been done with two cyanobacterial suspensions: *M. aeruginosa *QH/NR/Ma03, 5% w/v and *A. circinalis *non-toxic bloom sample, Gordonbrook Dam, Queensland, 10% w/v (lyophilised cyanobacteria in 75% methanol), with negative results [15]. Rietschel & Fowler [[16] (p. 15)] nominate appropriate steps for testing non-standard contactants: initial test concentrations of 0.1% to 1.0% performed on several volunteers, including the investigator. An autoexperiment was conducted on author IS in May 2001. Eight Finn chambers containing 5% w/v suspensions of *M. aeruginosa *QH/NR/Ma03 and the Gordonbrook Dam bloom sample containing predominantly *A. circinalis *were prepared; each suspension was applied with three vehicles: Milli-Q^® ^water, 50% v/v methanol in Milli-Q^® ^water, and acetone. Lyophilised, powdered *M. aeruginosa *and *A. circinalis *cells were each mixed in petrolatum and placed into two of the Finn chambers. Mild irritant reactions were seen on the aqueous *A. circinalis *suspension site, and on the two petrolatum sites. Because author IS has never suffered from dermatitis, we suspected that the irritant reaction, albeit mild, was probably the result of an artificially high concentration of cyanobacterial cells. So the maximum concentrations of cyanobacteria applied to the skin of volunteers (0.25% w/v) were 20-fold lower than the concentration that elicited a mild irritant reaction on the skin of author IS during pre-testing experiments; 0.25% is also 20 to 40-fold lower than concentrations that failed to elicit observable or measurable reactions on mouse ears during open application experiments for irritancy [15]. We did not proceed with using powdered, lyophilised cyanobacteria mixed in petrolatum because of the anticipated loss of precision in determining doses. It was elected to use aqueous cyanobacterial suspensions for these patch testing studies, as water is the solvent of choice in the vast majority of recreational settings, from which arise reports of acute cyanobacteria-related dermatoses. Concomitant exposure to ethanol can often be observed in Australian recreational environments, but not by the cutaneous route.

### General discussion

The findings of this small human study are that cutaneous reactions to cyanobacteria are infrequent, at least in the population we sampled and the dose ranges we used. The work in the accompanying paper by Stewart *et al *[15] complements this study, and demonstrates that purified cylindrospermopsin is capable of eliciting irritant and delayed-contact hypersensitivity reactions in mice. The small number of case and anecdotal reports in the literature also shows that cyanobacteria-associated dermatoses are infrequently reported, although mild, self-limiting illnesses, including pruritic rashes, are likely to be under-reported and under-diagnosed [[2,25] (p. 69)]. However, anecdotal reports of incident-free exposures to high levels of cyanobacteria have also been received [[10] (Chapter 4)]; author IS has tried without success to generate a cutaneous response on his own skin through open application of concentrated cyanobacterial cells on many occasions, from both field samples and laboratory isolates. Images of field workers demonstrating similarly enthusiastic disregard for occupational health and safety matters can be seen at [26-28].

The commercial sector has not been slow to realise that cutaneous responses to cyanobacteria are not unequivocally hazardous. A Google search using the terms "blue green algae" "soothes" and "skin" reveals a bewildering array of products and services that promise relief from much of what ails you. Many of these products are made from *Arthrospira *sp., a cyanobacterium also known as spirulina. Clinical and research dermatologists will no doubt be pleased to hear about:

**Spirulina wrap** : Rich in antioxidant vitamins, spirulina is the ultimate nutrient boost. This treatment stimulates and nourishes the skin while promoting a healthy, more vibrant appereance (*sic*). (50 minutes) [29]

So there is still a great deal to learn about cyanobacteria and the skin. To what degree these widespread organisms may affect the health of individuals with atopic and non-atopic allergic disease is unknown, but deserves the attention of researchers. The subject of photoallergy and photoirritancy has not been investigated. Most environmental exposures to aquatic cyanobacteria occur in recreational settings, which correlate strongly with exposure to sunlight, so photic effects should presumably be investigated.

Whether cyanobacteria-associated cutaneous eruptions in susceptible individuals are primarily irritant reactions, immediate hypersensitivity or delayed contact hypersensitivity responses is not at all clear. The picture may turn out to be complex and varied, with similarities to the broad topic of phytodermatitis. Wilkinson and Shaw [30] list the principal presenting features of phytodermatitis thus:

1. irritant contact phytodermatitis – both chemical and physical

2. allergic contact phytodermatitis – both immediate and delayed

3. phytophototoxic dermatitis

4. pseudophytophotodermatitis...

5. allergic contact phytodermatitis with secondary photosensitivity...

Cyanobacteria-related dermatoses may also operate through different molecular mechanisms and may therefore vary in clinical presentation via: individual susceptibility (e.g. atopic phenotype), cyanobacteria profile in waterbodies (different species, genera, cell biomass), cyanotoxins (different types, different mechanisms of toxicity, and variable concentration in waterbodies – i.e. exposure and dose concerns), disruption to barrier function from waterlogged skin, and the influence of ultra-violet irradiation (phototoxic effects or immunosuppressive?).

## Conclusion

This pilot study of 39 volunteers identified a single individual with atopic disease who responded to several cyanobacterial preparations applied to the skin by closed patch testing. Dose-response relationships were not observed in this individual, which supports the clinical findings that these were hypersensitivity reactions. This subject developed positive responses to all patch sites containing cylindrospermopsin, whereas none of the remaining 38 subjects showed any response to cylindrospermopsin. This work complements a mouse model study of delayed-contact hypersensitivity that demonstrates cylindrospermopsin is active in mammalian epidermal tissues. Future work into cutaneous effects of cyanobacteria in humans may benefit from improved awareness of cellular and molecular mechanisms to allow more refined targeting of higher-risk populations.

As case reports and epidemiologic studies do not present convincing findings of mass outbreaks of acute cutaneous responses to planktonic freshwater cyanobacteria, the possibility that many such reports are due to hypersensitivity reactions should be considered; these preliminary studies would seem to support this concept.

## Abbreviations

CSIRO Commonwealth Scientific and Industrial Research Organisation

i.p. intraperitoneal

LD_50 _lethal dose for 50% of test animals

LPS lipopolysaccharide

ppb parts per billion (μg/L)

ppm parts per million (mg/L)

PVC polyvinyl chloride

SDS sodium dodecyl sulfate (aka sodium lauryl sulfate)

## Competing interests

The author(s) declare that they have no competing interests.

## Authors' contributions

IS and IMR initiated the study concept and design. IS grew and processed cyanobacteria, co-ordinated the study, conducted statistical tests and drafted the manuscript. IMR conducted and supervised dermatologist patch test readings. PMW and PJS assisted with study design and statistical advice. GRS supervised the project. All authors read and endorsed the final manuscript.

## Pre-publication history

The pre-publication history for this paper can be accessed here:



## Figures and Tables

**Table 1:** Patch testing interpretation key

+/-	Uncertain reaction: faint macular erythema only
+	*Weak *(nonvesicular) positive reaction; erythema, infiltration, possibly papules
++	*Strong *(vesicular) positive reaction; erythema, infiltration, papules, vesicles
+++	*Extreme *positive reaction; bullous reaction
-	*Negative *reaction
IR	Irritant reaction of different types

Adapted from Rietschel & Fowler [16 (p. 24)] (interpretation key of the International Contact Dermatitis Research Group).

**Table 2:** Suspensions and extracts applied to patch test wells

**Test material or species**	**Patch series type**	**Strain**	**Source**	**Cyanotoxin (concentration in 0.25% w/v lyophilised cyanobacteria patch preparation)**
Sodium dodecyl sulfate (SDS, aka sodium lauryl sulfate)	Positive irritant control		Sigma-Aldrich P/L	
*Cylindrospermopsis raciborskii*	Cyanobacterial cell suspension	AWT 205 Non-axenic	Australian Water Technologies culture collection Sydney, Australia	Cylindrospermopsin (2.0 mg/L)
*C. raciborskii*	Cyanobacterial LPS extract	AWT 205 Non-axenic	Australian Water Technologies culture collection Sydney, Australia	
*Microcystis aeruginosa * *C. raciborskii * *Aphanizomenon *sp.	Cyanobacterial cell suspension		Field sample, North Pine Dam (South-east Queensland, Australia)	Microcystins (200 μg/L total microcystins expressed as MC-LR); cylindrospermopsin (6.4 μg/L)
*M. aeruginosa*	Cyanobacterial cell suspension		Field sample, Lake Coolmunda (Southern Queensland, Australia)	Non-toxic (nil detect for microcystins)
*M. aeruginosa*	Cyanobacterial LPS extract		Field sample, Lake Coolmunda (Southern Queensland, Australia)	
*M. aeruginosa*	Cyanobacterial cell suspension	QH/NR/Ma/03 Non-axenic	Queensland Health Scientific Services culture collection, Brisbane, Australia	Microcystins [predominantly microcystin-LR] (1.60 mg/L total microcystins expressed as MC-LR)
*Anabaena circinalis*	Cyanobacterial cell suspension		Field sample, Lake Coolmunda (Southern Queensland, Australia)	Saxitoxins (19 μg/L total saxitoxins expressed as saxitoxin)
*Planktothrix *sp.	Cyanobacterial cell suspension	QH/NR/Px/01 Non-axenic	Queensland Health Scientific Services culture collection, Brisbane, Australia	Non-toxic (nil detect for microcystins)
*Chlorella vulgaris*	Green algal cell suspension	CS-42 Non-axenic	CSIRO collection of living microalgae, Hobart, Australia	

**Table 3:** Summary of questionnaire responses: history of cutaneous and allergic illness.* n *(%)

	**Outpatients**			**Reference subjects**			
	
	**Yes**	**No**	**Not sure**	**Yes**	**No**	**Not sure**	**p**
*Eczema or dermatitis*							
Ever diagnosed	12(60)	4(20)	1(5)	7(37)	12(63)	0	0.04
Last two years	11(55)	3(15)	3(15)	5(26)	13(68)	0	0.01
*Asthma*							
Ever diagnosed	6(30)	13(65)	0	8(42)	10(53)	0	0.51
Last two years	5(25)	14(70)	0	5(26)	13(68)	0	1.0
*Hay fever*							
Ever diagnosed	2(10)	15(75)	1(5)	5(26)	14(74)	0	0.41
Last two years	3(15)	14(70)	1(5)	4(21)	15(79)	0	1.0
*Urticaria*							
Ever diagnosed	1(5)	17(85)	1(5)	2(11)	16(84)	0	1.0
Last two years	0	17(85)	1(5)	1(5)	16(84)	1(5)	1.0
*Rash of unknown cause*							
Last two years	10(50)	4(20)	3(15)	3(16)	15(79)	0	0.003
*Rash after freshwater recreation*	1(5)	16(80)	1(5)	0	15(79)	3(16)	1.0

n = 20 for the outpatient subject group; n = 19 for the reference group. Where sum of row answers (yes/no/not sure) is below the total, shortfall represents unanswered questions.

p-values: Fisher's exact test comparing proportion of "yes" and "no" answers between outpatient and reference subject groups

**Table 4:** Cyanobacterial and algal patch series: positive and equivocal patch test results

		**Subject**								
**Test material**	**Concentration**	**PT01**	**PTO2**	**PT04**	**PT06**	**PT19**	**CO05**	**CO06**	**CO08**	**CO09**

***C. raciborskii***	**0.005%**					(++)* (+)**				
**AWT 205 cell**	**0.05%**					(++)* (+)**				
**suspension**	**0.25%**					(++)* (+)**				
***C. raciborskii***	**630 ppb**							(+/-)*		
**AWT 205 LPS**	**6 ppm**									
**extract**	**31 ppm**									
***M. aeruginosa***										
***C. raciborskii***	**0.005%**					(+)* (+)**				
***Aphanizomenon *sp.**	**0.05%**					(++)* (+)**				
**North Pine Dam cell suspension**	**0.25%**					(+)* (+)**				
***M. aeruginosa***	**0.005%**					(+)**				
**Lake Coolmunda**	**0.05%**					(+)**				
**cell suspension**	**0.25%**				(+/-)*	(+/-)**				
***M. aeruginosa***	**260 ppb**							(+/-)*		
**Lake Coolmunda**	**3 ppm**								(+/-)*	(+/-)**
**LPS extract**	**13 ppm**				(+/-)*					(+/-)**
***M. aeruginosa***	**0.005%**						(+/-)*			
**QH/NR/Ma/03 cell**	**0.05%**									
**suspension**	**0.25%**		(+/-)*		(+/-)*					
***A. circinalis***	**0.005%**									
**Lake Coolmunda**	**0.05%**	(+/- IR)*			(+/-)*					
**cell suspension**	**0.25%**	(+ IR)*								
***Planktothrix *sp.**	**0.005%**									
**QH/NR/Px/01 cell**	**0.05%**	(+)*				(+)* (+)**				
**suspension**	**0.25%**					(+)**				
***C. vulgaris***	**0.005%**	(+/- IR)*		(+/-)*		(+)*				
**CS-42 cell**	**0.05%**			(+/-)*		(+)*	(+/-)*			
**suspension**	**0.25%**			(+/-)*		(+)*				

*: grading at 48-hour inspection

**: grading at 96-hour inspection

Subject prefix "PT" = dermatology outpatient subject

Subject prefix "CO" = non-patient volunteer

**Table 5:** Estimated doses of cyanotoxins by the cutaneous route

**Cyanotoxin**	**Dose per subject**	Dose by weight*	**Mouse LD**_50_**(i.p.)**
Cylindrospermopsin	160 ng	2.4 ng/kg	2.1 mg/kg (24 hours); 200 μg/kg (5–6 days) [31]
Microcystins	170 ng	2.6 ng/kg	45–70 μg/kg (most toxic forms) [32 (p. 140)]
Saxitoxins	3.8 ng	58 pg/kg	10–30 μg/kg [32 (p. 140)]

*Dose by weight estimated for a 65 kg individual
